# Co-infection of *Schistosoma mansoni*/hepatitis C virus and their associated factors among adult individuals living in fishing villages, north-western Tanzania

**DOI:** 10.1186/s12879-017-2780-3

**Published:** 2017-10-10

**Authors:** Humphrey D. Mazigo, Stella Kepha, Godfrey M. Kaatano, Safari M. Kinung’hi

**Affiliations:** 1 Department of Medical Parasitology and Entomology, School of Medicine, Catholic University of Health and Allied Sciences, P.O. Box 1464, Mwanza, Tanzania; 2 Pwani University, Kilifi, 195-80108 Kenya; 3 National Institute for Medical Research, Mwanza Research Centre, Mwanza, Tanzania

**Keywords:** *S. mansoni*, Hepatitis C, Co-infection, Hepatomegaly, Splenomegaly, Periportal fibrosis, Fishing village, Tanzania

## Abstract

**Background:**

*Schistosoma mansoni* and Hepatitis C virus (HCV) are co-existence in sub-Saharan Africa and co-infection is common among humans population. The immunological responses characterized with Th_2_-immune responses for *S. mansoni* and Th_1_-immune responses for HCV are responsible for development hepatic morbidities in infected individuals. However, the co-occurrences of *S. mansoni* and HCV infection, their related ultrasound detectable morbidities and associated risk factors at community levels have not been examined in fishing communities, north-western Tanzania. In this context, the present study covers that gap.

**Methods:**

A cross-sectional study was conducted among 1924 asymptomatic individuals aged 15–55 years in four fishing villages (Igombe, Igalagala, Sangabuye and Kayenze) of Northwestern Tanzania. A single stool sample was collected from each study participants and examined for *S. mansoni* eggs using Kato Katz technique. Hepatitis C surface antigen (HCVsAg) was determined from a finger prick blood sample using a rapid test.

**Results:**

Overall, 51.8% (997/1924; 95%CI: 49.6–54.1) of the study participants were infected with *S. mansoni* and had a mean intensity of 223.7epg (95%; 202.4–247.1). Of the study participants, 90 (4.7%) were infected with hepatitis C virus (HCV). Overall, 2. 4% (47/1924) of the study participants were co-infected with *S. mansoni* and hepatitis C virus. Among the co-infected individuals, 42.6%, 70.2% and 19.1% had splenomegaly, hepatomegaly and periportal fibrosis (PPF). Factors associated with *S. mansoni*/HCV co-infection were being aged 26–35 years (aRR = 2.67, 95%CI: 1.03–6.93, *P* < 0.04), 46–55 years (aRR = 2.89, 95%CI: 1.10–7.57, *P* < 0.03) and having marked hepatomegaly (aRR = 2.32, 95%CI: 1.09–4.9, *P* < 0.03).

**Conclusion:**

In this setting, *S. mansoni* and Hepatitis C are co-endemic and a proportion of individuals were co-infected. Hepatosplenic morbidities characterized with hepatomegaly, splenomegaly, hepatosplenomegaly and PPF were observed in co-infected individuals. These results highlight the need for integrated interventions measures against parasitic and viral diseases.

## Background

Intestinal schistosomiasis caused by *Schistosoma mansoni* infection remains one of the public health concerns in Tanzania [1]. This infection is highly endemic in fishing villages bordering the southern shore and islands within the Lake Victoria [1–3]. One third of the estimated 44 million Tanzanian populations are estimated to be infected with *S. mansoni* [1, 4]. Chronic *S. mansoni* infection is mainly associated with deposition of eggs of the parasites in human body tissues especially the liver [5, 6]. Immunological responses against trapped eggs are responsible for the development of granulomas, chronic exposure to which may end with development of hepatosplenic schistosomiasis [5, 6]. Hepatosplenic morbidities are characterized by hepatic and splenic enlargement, progressive periportal fibrosis (PPF) which can lead to portal hypertension and its associated sequelae such as liver surface irregularities and portal-systemic venous, with the risk of esophageal varices and haemetemesis [5–7].

Conversely, hepatitis C virus (HCV) infection is endemic in sub-Saharan Africa and its prevalence varies from one epidemiological setting to another [8, 9]. Worldwide, 185 million individuals are estimated to be infected with HCV based on antibodies testing, with the highest prevalence observed in Asia and Middle East [10]. In sub-Saharan Africa, the prevalence of HCV infection ranges from 1% in Ethiopia to 50% in Egypt [10, 11]. It is estimated that, 70% of the individuals infected with HCV go on to develop chronic hepatitis and an approximate of 15–20% of them develop cirrhosis [12, 13].

Because of co-endemicity of *S. mansoni* and HCV infection in sub-Saharan Africa, co-infection does occur, especially in areas where intestinal schistosomiasis is highly endemic [9, 14, 15]. The pathogenesis of *S. mansoni* and HCV in co-infected individuals on liver fibrosis is well established [5, 11–13]. However, the pathomechanism of the two infections differs by the fact that *S. mansoni* immunological responses are mainly characterized by Th_2_-CD4^+^ type immune responses [5]; whereas for HCV infection, a type 1 Th_1_-CD8^+^ like responses is thought to play role in hepatic inflammatory responses and viral clearance [16]. Because of the immunological differences, the characteristics of hepatic damage resulting from the two infections also differs [5, 12, 13], with HCV infection causing advanced liver disease, increased incidence of liver cirrhosis and hepatocellular carcinoma [12, 13, 16].

In Tanzania, *S. mansoni* [1] and HCV infections [17–19] are co-endemic. It is estimated that 52% of the Tanzanian population are infected with schistosomiasis (both *S. mansoni* and *S. haematobium*) [4]. In Tanzania, *S. haematobium* is widely distributed, whereas *S. mansoni* is mainly restricted to large water bodies and paddy farming areas [20]. *Schistosoma mansoni* is common in areas surrounding the Lake Victoria shores and islands within the lake [2, 21, 22]. To date, there are no data on the national prevalence of HCV infection and information from the global burden of diseases indicate that, the annual mortality rate per 100,000 people from Hepatitis C in Tanzania has increased by 17.4% since 1990, an average of 0.8% a year [23]. Despite the fact that the two infections are co-endemic in the country, no community study has studied their co-occurrences and their related morbidities, especially in fishing communities. To justify the public health aspects of integrating interventions measures against parasitic and viral diseases at community level, the aim of the present study was to determine the prevalence of *S. mansoni* and hepatitis C co-infections, related hepatosplenic morbidities and associated risk factors among study participants.

## Methods

### Study area

The current data are from the secondary analysis of the study that was conducted in northwestern Tanzania [7, 24]. Briefly, the study was conducted in fishing villages of Ilemela district, including the villages of Igombe, Igalagala, Sangabuye and Kayenze bordering the Lake Victoria shore on the southern part at Ilemela district, Mwanza region (32-34°E and 2-4^0^S, north-western Tanzania. The area experiences a temperature range from 18 °C to 28 °C and the mean annual rainfall of 1068 mm. The majority of the inhabitants of these villages are involved in farming and fishing activities. Because of high water contact levels, inhabitants remain at high risk of being infected with *S. mansoni* [2]. The control of *S. mansoni* infection in the study area involves mass chemotherapy using praziquantel drug, which is mainly focused in school children to reduce the long-term morbidities associated with the infection. There is no any program focusing on HCV control, screening and treatment at community.

### Study design, inclusion and exclusion criteria

This was an analytical cross-sectional study conducted between September 2012 – December 2012 and the data presented in current work are secondary analysis of the work published elsewhere [7, 24]. The study included participants (i) aged 15–55 years old and (ii) lived in the study villages for more than 2 years (iii) with no history of HCV infection. Study participants with history of treatment against *S. mansoni* infection (praziquantel drug) in the past 6 month, on anti-HCV treatment and clinical diagnosed with HCV infection (advised to seek medical care at the district hospital) were excluded from the study.

### Sample size, sampling technique and recruitment

Sample size calculation and sampling procedures have been previously described in details in Mazigo et al.,. [24]. Briefly, a total of 2142 study participants were enrolled into the study of which 1924 were eligible for the final analysis after fulfilling inclusion criteria. A two-step sampling procedures was used to select households and household members to participate in the study. A list of households and household members was obtained and from this, a random sampling procedure was used to select eligible individuals from randomly selected households.

### Data collection

#### Parasitological examination of stool for *Schistosoma mansoni* and *S. haematobium* eggs

A single stool and urine samples was collected from all consented study participants using labeled clean containers. From the stool sample, four Kato Katz thick smears were prepared from different sites of each stool sample using a template of 41.7 mg (Vestergaard Frandsen, Lausanne, Switzerland), following a standard protocol [25]. In brief, four Kato-Katz thick smears were prepared on microscope slides and labeled with a participant identification number. After 24 h, the smears were examined for *S. mansoni* eggs independently by two experienced laboratory technician at the laboratory of the National Institute for Medical Research, Mwanza. The number of eggs for *S. mansoni* were counted and recorded separately in the prepared parasitological forms. For quality assurance, a random sample of 10% of the negative and positive Kato Katz thick smears were re-examined by a third technician. For diagnosis of *S. haematobium* infection, collected urine samples were examined grossly for presence of macrohaematuria and using Hemastrix dipstick for presence of microhaematuria. Then, all urine samples were further examined using urine filtration technique with Nuclepore® membrane according to WHO [26].

#### Examination of hepatitis-C virus infection

For diagnosis of hepatitis- C virus infection, the finger prick blood sample was collected from each study participants and used for screening of hepatitis-C infection using a qualitative rapid test for detection of hepatitis C antigen in whole blood, (ACON Laboratories, Inc., San Diego, CA) according to manufacturer’s instructions at the field site laboratory [27]. Briefly, a drop of blood sample was added in the test strip followed by drop of the provided buffer. The results of the test were read after 15–20 min. The test was regarded as positive if the control and test line appeared after the given time. The test was regarded negative if only a single control line appeared.

#### Screening for human immunodeficiency Virus-1

The Tanzanian National HIV algorithms which recommended (currently has been revised) the use of a rapid test Determine (Alere Determine, Chiba, Japan) and Uni-GOLD (Trinity Biotech PLV, Bray, Ireland) was used for HIV diagnosis. Participants were counseled before and after HIV testing as per recommendation [28].

#### Ultrasonographical examination of hepatosplenic morbidities

All study participants were examined clinically for presence of any organomegally (enlargement of the liver and spleen) [29]. Two medically personnel trained on Niamey protocol examined study participants using a portable ultrasound machine (Aloka, Tokyo) [30]. Identified pathology was classified as per modified Niamey protocol [30]. The liver texture patterns, peripheral portal branches (PPBs), periportal fibrosis (PPF), thickness of PPB walls, spleen size, splenic vein (SV) diameter and ascites were assessed. Periportal fibrosis (PPF) was defined according to WHO [15] and the degree of PPF was categorized as A, B, C, D, E and F [30]. Periportal fibrosis grade A and B were classified as normal.

### Data analysis

A CSPro system was used for double data entry and data analysis was performed using Stata Version 12 (Stata Corp, college station, Texas, USA). Categorical variables were summarized by numbers and percentages. Comparison of proportions/categorical variables was done using chi-square (χ^2^)/fisher exact where appropriate. For continuous variables descriptive statistics were reported as means with standard deviation for normally distributed variable and medians with interquartile ranges (IQR) for variables that were not normally distributed. The arithmetic mean of *S. mansoni* egg counts for each participant was calculated from the counts of four Kato Katz thick smears and multiplied by 24 to obtain individual eggs per gram of faeces. *Schistosoma mansoni* egg counts were logarithmically transformed to allow calculation of the geometric mean egg per gram of feaces (GM-epg), which was calculated as an antilog of the mean of the transformed egg counts. Geometric mean egg counts for *S. mansoni* between sex and age were compared using Student-t-test (two groups) or ANOVA (more than two groups). Intensity of infection was categorized according to WHO criteria as: 1–99 epg, 100–399 epg, ≥400 epg defined as low, moderate and heavy intensities of infection respectively [31].

The categorization of the ultrasound measurements was based on the Niamey protocol [30]. To identify factors associated with *S. mansoni*/HCV co-infection, binomial regression model was constructed. At bivariate analysis, factors with *P*-values of 0.2 were considered for multiple binomial regression analysis. Because PPF grades, splenomegaly and left liver lobe hepatomegaly were related, only left liver lobe hepatomegaly was considered for multiple binomial regressions. A *P*-value of <0.05 was considered significant.

### Ethical consideration

Ethical approval was sought from the Research and Ethics Committees of Bugando University College of Health Sciences and Allied Sciences-Institutional Review Board, (BREC/001/32/2011). Ethical clearance was granted by the National Ethical Review Committee, National Institute for Medical Research, Tanzania. Swahili translated informed assent and consent forms were used to obtain children and adult participants’ consent respectively. For illiterate individuals, a thumb print was used to sign the assent and consent forms after a clear description of the study objective was explained to them and accepted to participate.

## Results

### Demographic characteristics of the study participants

**Fig. 1:**
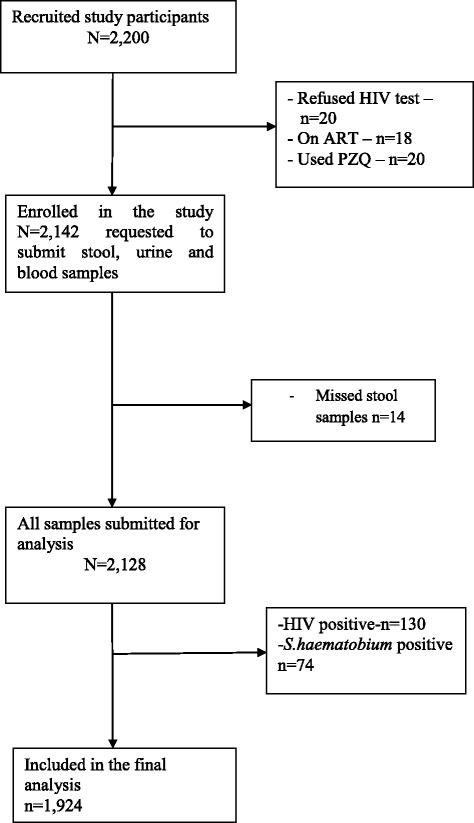
Flow chart detailing participation and adherence of study participants in the study

A total of 1924 study participants aged 15–55 years were enrolled into the present study (Fig. 1). Of these participants, 46.7% (*n* = 899/1924) and 53.3% (*n* = 1025) were male and female respectively. The mean age of the study participants was 32.75 ± 11.15 years. The main economic activities of the study participants were farming (72.5%, *n* = 1396), fishing (13.9%, *n* = 268) and small-scale business (13.5%, *n* = 260). Overall, 33.3% (*n* = 638) and 66.7% (*n* = 1284) of the study participants were illiterate and literate respectively. Table 1 shows demographic characteristics of the study participants.

**Table 1:** Demographic characteristics of the study participants

Characteristics	*N*	Sex		χ^2^	*P*-value
		Female *n*(%)	Male *n*(%)		
Age groups (in years)					
15–25	584	330(57.2)	250(43.1)	6.9748	0.01
26–35	553	272(49.2)	281(50.8)		
36–45	385	209(54.3)	176(45.7)		
46–55	402	212(52.7)	190(47.3)		
Village of residence					
Sangabuye	482	259(53.7)	223(46.3)	10.3077	0.02
Kayenze	708	378(53.4)	330(46.6)		
Igombe	456	262(57.5)	194(42.5)		
Igalagala	278	126(45.3)	152(54.7)		
Education level					
Literate	1284	614(47.8)	670(52.2)	52.4112	0.001
Illiterate	638	418(65.5)	220(34.5)		
Occupation					
Small scale business	260	187(71.9)	73(28.1)	291.1728	0.001
Farming	1396	827(59.2)	569(40.8)		
Fishing	268	22(8.0)	247(92.1)		

### Prevalence and intensity of *Schistosoma mansoni* infection

The overall prevalence of *S. mansoni* was 51.8% (997/1924, 95%CI: 49.6–54.1) with male in individuals having the highest prevalence compared to female individuals (44.1% versus 60.6%, *P* < 0.001) (Table 2). Similarly, the youngest age group (15–25 years) having the highest prevalence compared to older age groups (χ^2^ = 77.0276, *P* < 0.001). In relation to village of residence, study participants from Igalagala and Kayenze villages had the highest prevalence of *S. mansoni* infection than participants from other participating villages (χ^2^ = 42.4633, *P* < 0.001). Similarly, in relation to occupation, participants reported to be involved in fishing activities had the highest prevalence compared to study participants involved in farming and small scale business (χ^2^ = 30.8409, *P* < 0.001) (Table 2).

**Table 2:** Prevalence of *Schistosoma mansoni* in relation to demographic characteristics of the study participants

Characteristics	*N*	*Schistosoma mansoni*		χ^2^	*P*-values
		Negative	Positive		
Sex					
Male	899	354(39.4)	545(60.6)	52.3860	0.001
Female	1025	573(55.9)	452(44.1)		
Age groups (in years)					
15–25	584	211(36.1)	373(63.9)	77.0276	0.001
26–35	553	253(45.7)	300(54.3)		
36–45	385	225(58.4)	260(41.6)		
46–55	402	242(60.2)	160(39.8)		
Village of residence					
Sangabuye	482	264(54.8)	218(45.2)	42.4633	0.001
Kayenze	708	290(40.9)	418(59.1)		
Igombe	456	259(56.8)	197(43.2)		
Igalagala	278	114(41.0)	164(58.9)		
Occupation					
Small scale business	260	142(54.6)	118(45.4)	30.8409	0.001
Farming	1396	712(51.0)	684(48.9)		
Fishing	268	196(35.8)	172(64.2)		

The overall, Geometrical mean egg per gram of feaces (GMepg) was 223.7(95%CI: 202.4–247.1, range: 24-30192epg), with male individuals having the highest infection intensity (*t* = −4.7597, *P* < 0.001). Similarly, the youngest age group had the highest infection intensity (F = 1.62, *P* < 0.001).

### Seroprevalence of hepatitis C virus infection

The overall prevalence of Hepatitis C virus (HCV) infection was 4.7% (90/1924, 95%CI: 3.7–5.7). There was no sex difference in prevalence of HCV (χ^2^ = 0.0001, *P* = 0.99) (Table 3). Age difference in prevalence of HCV infection was observed with the oldest age group having the highest prevalence (χ^2^ = 25.0304, *P* < 0.001). Similarly, study participants from Kayenze village recorded the highest prevalence of HCV infection compared to participants from other participating villages (χ^2^ = 11.9520, *P* < 0.01).

**Table 3:** Prevalence of hepatitis C virus in relation to demographic characteristics of the study participants

Characteristics	*N*	Hepatitis C virus infection		χ^2^	*P*-value
		Negative	Positive		
Sex					
Male	1025	977(95.3)	48(4.7)	0.0001	0.99
Female	899	857(95.3)	42(4.7)		
Age groups (in years)					
15–25	584	575(98.5)	9(1.5)	25.0304	0.001
26–35	553	522(94.4)	31(5.6)		
36–45	385	368(95.6)	17(4.4)		
46–55	402	369(91.8)	33(8.2)		
Village of residence					
Sangabuye	482	462(95.9)	20(4.2)	11.9520	0.01
Kayenze	708	662(93.5)	46(6.5)		
Igombe	456	446(97.8)	10(2.2)		
Igalagala	278	264(94.9)	14(5.0)		

### Co-infection of *Schistosoma mansoni* and hepatitis C virus

The overall prevalence of co-infection of *S. mansoni* and hepatitis C was 2.4% (47/1924). The overall GMepg of the study participants who were co-infected with *S. mansoni*/HCV infection was 206.95GMepg (95%CI: 128.4–333.6). Among the co-infected, a sex difference was observed with male individual’s having the GMepg of 225.9epg (95%CI; 106.9–477.1) and for female individuals had GMepg of 188.9epg (95%CI: 98.9–360.6).

### Hepatosplenic morbidities in *S. mansoni* and hepatitis C virus co-infected individuals

Among the study participants who were infected with hepatitis C virus (*n* = 90), 45.6% (*n* = 41) and 20% (*n* = 18) had enlarged and marked enlarged left liver lobe (left liver lobe hepatomegaly). For splenomegaly, 23.3%(21/90) and 10%(9/90) of the study participants had moderate and severely enlarged spleen. In relation to co-infection, study participants who were co-infected with *S. mansoni* and HCV (*n* = 47), 44.7% (21/47) and 25.5% (12/47) had moderate and marked enlarged left liver lobe. For the splenomegaly, 27.7% (13/47) and 14.9% (7/47) of the co-infected study participants had moderate and severely enlarged spleen. Similarly, 36.2% (*n* = 17/47) of the co-infected individuals had hepatosplenomegaly (enlarged both left liver lobe and spleen).

Overall, the prevalence of periportal fibrosis (PPF) among study participants infected with HCV was 15.6% (14/90) (PPF grades, C = 2, D = 11 and E-F = 1). For co-infected study participants (n = 47), 19.1% (9/47) had PPF (PPF grades, C = 1, D = 7 and E-F = 1).

### Factors associated with *Schistosoma mansoni* and Hepati*t*is C virus co-infections

At bivariate analysis, age groups 26–35 years and 46–55 years, living at Kayenze village, having marked left liver lobe hepatomegaly, having splenomegaly and PPF grade D were associated with *S. mansoni* and hepatitis C virus co-infection (Table 4). At multiple binomial regression analysis, belong to the age group 26–35 years (aRR = 2.67, 95%CI: 1.03–6.93, *P* < 0.04) and 46–55 years (aRR = 2.89, 95%CI: 1.10–7.57, *P* < 0.03), living at Kayenze village (aRR = 3.67,95%CI: 1.62–8.31, *P* < 0.002) and having marked hepatomegaly (aRR = 2.32,95%CI: 1.09–4.91, *P* < 0.03) remained independently associated with co-infection of *S. mansoni* and hepatitis C virus infections (Table 4).

**Table 4:** Risk factors associated with *S. mansoni* and Hepatitis C virus infections among individuals living in fishing villages of northwestern Tanzania

Variable	cRR	95%CI	*P*-value	aRR	95%CI	*P*-value
Sex						
Female	1			1		
Male	1.66	0.9–2.9	0.1	1.45	0.8–2.7	0.24
Age groups (in years)						
15–25	1			1		
26–35	3.18	1.2–8.3	0.02	2.67	1.0–6.9	0.04
36–45	0.77	0.2–2.8	0.69	0.83	0.2–3.1	0.79
46–55	3.06	1.2–8.1	0.02	2.89	1.1–7.6	0.03^a^
Occupation						
Small scale business	1			1		
Farming	1.58	0.6–4.4	0.9	1.31	0.5–3.6	0.6
Fishing	2.93	0.9–9.4	0.1	1.89	0.6–6.3	0.3
Village of residence						
Sangabuye	1			1		
Kayenze	3.53	1.6–7.9	0.002	3.67	1.6–8.3	0.02^a^
Igombe	0.57	0.16 – 1.9	0.37	0.66	0.2–2.3	0.51
Igalagala	2.26	0.8–6.3	0.12	2.14	0.8–5.9	0.14
Education level						
Literate	1			–	–	–
Illiterate	1.18	0.66–2.0	0.58	–	–	–
Left liver lobe hepatomegaly						
Normal	1			1		
Moderate	1.60	0.8–3.1	0.16	1.79	0.9–3.5	0.1
Marked	2.26	1.1–4.8	0.03	2.32	1.1–4.9	0.03^a^
Splenomegaly						
Normal	1			–	–	–
Moderate	1.86	0.9–3.5	0.05	–	–	–
Marked	2.97	1.4–6.5	0.01	–	–	–
Periportal fibrosis (PPF) grades						
Normal	1					
Grade C	0.54	0.1–3.8	0.54	–	–	–
Grade D	2.39	1.1–5.1	0.03	–	–	–
Grade E & F	1.02	0.1–7.1	0.98	–	–	–

^a^Significant factors, *cRR* Crude Risk Ration, *aRR* Adjusted Risk Ratio

## Discussion

The findings of the present study on *S. mansoni* infection confirms the report of the previous reports in northwestern Tanzania [2, 3, 32], which demonstrated high prevalence of *S. mansoni* and infection intensity among adult population [2, 3]. Furthermore, our findings confirm high infection intensity among male individuals and young age groups [2, 3]. The variation in exposure to risk areas such as the lake and time spent in water sources (lake) partly explain the observed differences in prevalence and infection intensity between sex and between age groups [33–35]. In addition, a noted difference in prevalence of *S. mansoni* infection between villages was observed. Partly, this observation is explained by geographical location of the villages from the Lake Victoria shores, with Kayenze and Igalagala villages are located at the shoreline of the lake [2]. The geographical variations of *S. mansoni* infection even in villages located in the same area have been noted elsewhere in Africa [6, 33, 35].

Our findings noted a low prevalence of HCV infection among adult population in fishing communities. Population based studies in health adult individuals have reported a prevalence of 0.7% of HCV infection in Dar Es Salaam [36] and 1.2% in Northwestern Tanzania based on antibodies detection using ELISA technique [18]. In special groups such as blood donors, lower prevalence of HCV infection (1.5%) was reported in Dar Es Salaam, Tanzania [19]. Similarly, a lower prevalence of HCV antibodies (1.2%) was reported among health workers [37]. In contrast, a high seroprevalence of HCV infection (57% based on HCV antibody) was reported among a cohort of opioid treatment patients in Dar Es Salaam, Tanzania [38]. Compared to findings from other African countries, a prevalence of 8.1% [39], 12.8% [40] and 13.7% [41] of anti-HCV infection has been reported in Angola Nigeria and Democratic Republic of Congo. The observed variation in prevalence among different groups mainly reflects different levels of exposure to risk factors [38]. Hepatitis C virus infection is transmitted predominantly through exposure to contaminated blood and body fluids, thus, groups of individuals with high exposure to body fluids such as health workers and drug abusers always present with high prevalence of HCV infection [12, 38].

Our analysis on prevalence of HCV infection noted age difference in the prevalence of this viral infection. The older age groups (≥36 years) had the highest prevalence of HCV infection. The report of global burden of diseases indicates that hepatitis C virus infection is more common in the older age groups (75–79 years) in Tanzania [23]. This observation depicts the chronicity of the viral infection [12, 13]. Perhaps, the infection starts at young ages and its related morbidities and mortality are observed at older age groups [12].

In the present study a proportion of study participants were co-infected and had different patterns of hepatosplenic morbidities related to *S. mansoni* and Hepatitis C virus infection. Similar findings have been observed in endemic areas of sub-Saharan Africa, where both *S. mansoni* and HCV are co-endemic [8–10, 14]. However, the prevalence of co-infection of *S. mansoni* and HCV observed in the present study was comparable to findings of similar study in Ethiopia (4%) [15] and lower than what was observed in Egypt (33% and 40.2%) [42, 43]. Specifically, in Egypt, the prevalence of HCV is very high compared to many of the sub-Saharan African countries [8, 10], thus, co-infection of *S. mansoni* and HCV is also very high [8, 10]. It is worthwhile noting that, the present study used rapid diagnostic test to detected hepatitis C surface antigens (HCVsAg) which may have lower sensitivity than HCV antibodies detection methods used in previous studies [44]. Perhaps in these setting, the prevalence could be very high if HCV antibodies detection method could have been used. This is an open area calling for further studies using the more reliable diagnostic technique with improved sensitivity and preferably with a large sample size to establish the associated effects.

In the present study, study participants had hepatosplenic morbidities characterized by hepatic and splenic enlargements and periportal fibrosis characterized with different grades. Similar observations with different PPF grades and hepatosplenomegaly have been reported by previous studies [2, 3]. In co-infected individuals studies have shown that, *S. mansoni* infections increases HCV morbidities and chronicity of the liver pathology [45], increases HCV RNA titres, incidence of cirrhosis/hepatocellular carcinoma and higher mortality rates [45]. The effects of *S. mansoni* infection in HCV infected patients have been reviewed in details elsewhere [8]. However, the role of *S. mansoni* infection in exacerbating hepatic morbidities related to HCV infection remain a topic of debate, with other studies reporting no evidence that *S. mansoni* infection affect the outcome of HCV in infected individuals [46]. Given the low prevalence of HCV infection observed in the present study population, this HCV infection is likely to account for only a small proportion of chronic liver disease in the studied population. In addition, it is difficult to draw a solid conclusion on the role of *S. mansoni* infection in HCV co-infected patients in a study which had only 17 study participants who were co-infected and had hepatosplenic morbidities [45]. Large sample sizes will be needed in further studies to add more evidence.

The main risk factors associated with *S. mansoni*/HCV infection were mainly being of older age group (46–55 years), village of residence and having left liver lobe hepatomegaly. The association of these infections with older ages indicates that, these are chronic infections, which needs time for their obvious related morbidities such as hepatomegaly and fibrosis/liver cirrhosis to manifest [6, 12, 13]. The association with village of residence mainly defines the presence of exposure factors in these villages [7, 15]. Individually, epidemiological studies have shown that, *S. mansoni* infection is associated with village of residence, age, occupation, specifically fishing, being male and hepatosplenic morbidities [1, 6, 7, 47]. Older age groups, being male and residing in rural areas have been demonstrated to be associated with HCV infection [11].

The present study was subject to limitation, being a cross-sectional study in nature, partly may contribute to lack of temporal association between the *S. mansoni*/HCV as the main outcomes with some of the study variables. Also, the use of only one diagnostic technique to diagnosed HCV infection (HCVsAg), partly, may have underestimated the prevalence of HCV infection in the study population. In addition, the use of a single stool sample to examine for *S. mansoni* infection owing the day to day variability of parasite eggs output and low sensitivity of the Kato Katz technique in detecting individuals with light infection intensity may have under estimated the prevalence of *S. mansoni* infection in the studied population.

## Conclusion

The present study setting is endemic to *S. mansoni* and Hepatitis C virus infections and a small proportional of individuals are co-infected. *Schistosoma mansoni* related PPF grades, hepatomegaly, splenomegaly and hepatosplenomegaly were also present in co-infected individuals. Co-infection of *S. mansoni*/HCV was mainly associated with older age, village of residence and having hepatomegaly. Further studies are recommended in these areas to understanding the impact of *S. mansoni*/HCV co-infection before implementation of integrated interventions measures can be thought.

## Abbreviations

ARR: Adjusted Risk Ratios
cRR: Crude Odd Ratio
Epg: Egg per Gram of Feaces
GMepg: Geometrical Mean egg per gram of feaces
HCV: Hepatitis C Virus
HCVsAg: Hepatitis C Virus surface antigen
Mg: Milligram
PPF: Periportal fibrosis
Th: Lymphocytes type T- helper
